# Fertility in male mice lacking NO-sensitive guanylyl cyclase

**DOI:** 10.1186/1471-2210-11-S1-P30

**Published:** 2011-08-01

**Authors:** Dieter Groneberg, Peter König, Andreas Friebe

**Affiliations:** 1 Physiologisches Institut, Universität Würzburg, Germany; 2 Institut für Anatomie, Universität Lübeck, Germany

## Background

The NO/cGMP signal transduction is involved in the regulation of a variety of physiological processes e.g. smooth muscle relaxation, inhibition of platelet aggregation and synaptic plasticity. Nitric oxide (NO) is produced by NO synthases and acts mainly on NO-sensitive guanylyl cyclase (NO-GC), the most important NO receptor. NO stimulation of NO-GC leads to production of the second messenger cGMP which exerts its effects via cGMP-dependent kinases, channels or phosphodiesterases.

## Methodology

Recently, we have generated mice lacking NO-GC (GCKO). We showed that lack of NO-GC resulted in arterial hypertension concomitant with a totally abolished NO responsiveness of vascular and gastrointestinal smooth muscle.

Global deletion of a protein in mice does not allow identification of the cell/tissue type responsible for a certain phenotype. We therefore generated a mouse line in which NO-GC was specifically deleted in smooth muscle cells (SM-GCKO). These mice should provide more detailed information on the role of NO and cGMP with regards to smooth muscle relaxation.

## Results

Here we examined the role of NO/cGMP signaling with regards to the smooth muscle relaxation of corpus cavernosum. NO failed to relax corpus cavernosum from GCKO in organ bath experiments: neither exogenously produced NO by NO donors nor endogenous NO release from neurons induced by electrical field stimulation led to relaxation. Similar results were observed in the corpus cavernosum of SM-GCKO mice. To our surprise, both KO models were fertile and produce offspring.

## Conclusion

Our data show that deletion of NO-GC globally or exclusively in smooth muscle abolishes corpus cavernosum relaxation, but nevertheless, does not impair fertility.

